# Prediction of left lobe hypertrophy after right lobe radioembolization of the liver using a clinical data model with external validation

**DOI:** 10.1038/s41598-022-25077-6

**Published:** 2022-12-01

**Authors:** Jens M. Theysohn, Aydin Demircioglu, Malte Kleditzsch, Johannes M. Ludwig, Manuel Weber, Lale Umutlu, Yan Li, Malte Kircher, Constantin Lapa, Andreas Buck, Michael Koehler, Moritz Wildgruber, Christian M. Lange, Xavier Palard, Etienne Garin, Ken Herrmann, Michael Forsting, Felix Nensa

**Affiliations:** 1 Department of Diagnostic and Interventional Radiology and Neuroradiology, University Hospital Essen, University of Duisburg-Essen, Hufelandstrasse 55, 45122 Essen, Germany; 2 Division of Interventional Radiology, Department of Radiology and Biomedical Imaging, Yale School of Medicine, 330 Cedar Street, New Haven, CT 06510 USA; 3 Clinic for Nuclear Medicine, University Hospital Essen, University of Duisburg-Essen, Essen, Germany; 4 Nuclear Medicine, Medical Faculty, University of Augsburg, Augsburg, Germany; 5 Clinic for Nuclear Medicine, University Hospital Wuerzburg, Wuerzburg, Germany; 6 Radiology, University Hospital Muenster, Muenster, Germany; 7 Department for Radiology, University Hospital, LMU, Munich, Germany; 8 Clinic for Gastroenterology and Hepatology, University Hospital Essen, University of Duisburg-Essen, Essen, Germany; 9 Department of Nuclear Medicine, Centre Eugène Marquis, Rennes, France; 10 Institute for Artificial Intelligence in Medicine, University Hospital Essen, University of Duisburg-Essen, Essen, Germany

**Keywords:** Hepatocellular carcinoma, Predictive markers

## Abstract

In cirrhotic patients with hepatocellular carcinoma (HCC), right-sided radioembolization (RE) with Yttrium-90-loaded microspheres is an established palliative therapy and can be considered a “curative intention” treatment when aiming for sequential tumor resection. To become surgical candidate, hypertrophy of the left liver lobe to > 40% (future liver remnant, FLR) is mandatory, which can develop after RE. The amount of radiation-induced shrinkage of the right lobe and compensatory hypertrophy of the left lobe is difficult for clinicians to predict. This study aimed to utilize machine learning to predict left lobe liver hypertrophy in patients with HCC and cirrhosis scheduled for right lobe RE, with external validation. The results revealed that machine learning can accurately predict relative and absolute volume changes of the left liver lobe after right lobe RE. This prediction algorithm could help to estimate the chances of conversion from palliative RE to curative major hepatectomy following significant FLR hypertrophy.

## Introduction

In patients with hepatocellular carcinoma (HCC) and compensated liver cirrhosis, partial hepatectomy is one of the most valuable curative treatment options available^1^. However, when major hepatectomy is needed for complete tumor resection, many patients are not suitable for this procedure due to small future liver remnant (FLR) carries the risk of post-hepatectomy liver failure. In these patients, homolateral portal vein embolization (PVE) can be performed to induce hypotrophy of the embolized and compensatory hypertrophy of the contralateral liver lobe, thereby increasing the liver remnant (FLR) prior to resection^2^. However, as the tumor remains untreated, PVE imposes the risk of intermittent tumor progression leading to non-resectability in ~ 8% of cases^3^. Adding sequential transarterial chemoembolization to PVE can reduce this risk^4^.

**Figure 1:**
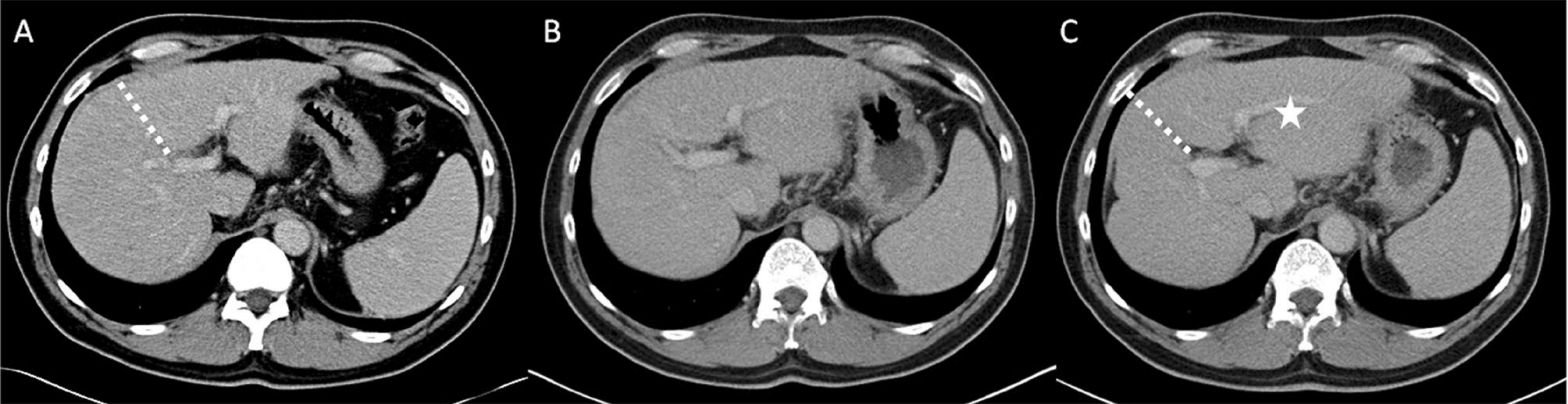
Hypertrophy of the left liver lobe after right-sided radioembolization. Computed tomography images (**A**) pre, (**B**) 3, and (**C**) 6 months after radioembolization. Significant volume increase of the left liver lobe (*) can be observed post-therapy. Dotted line: border between left and right liver lobe.

**Figure 2:**
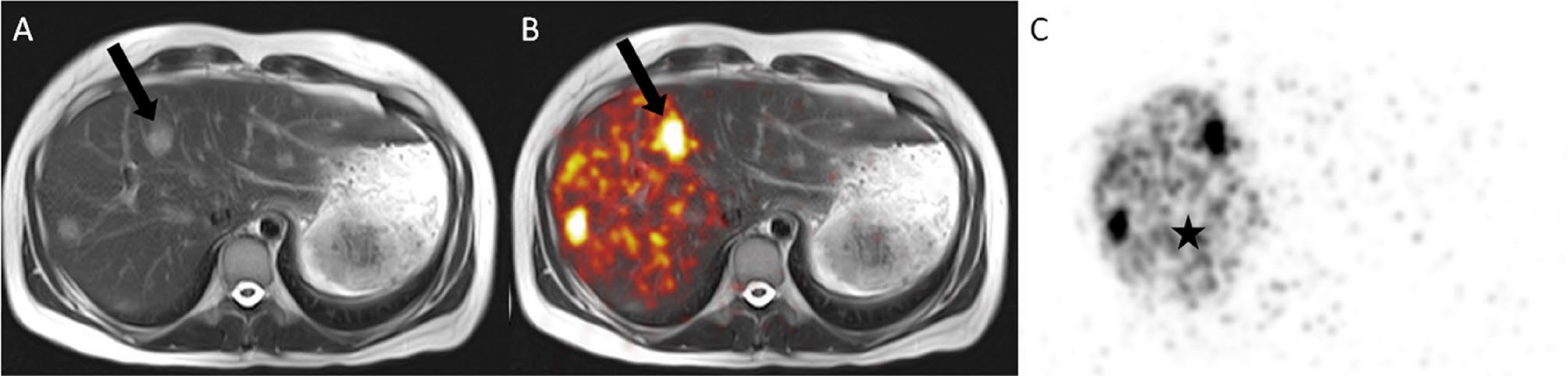
PET/MR after radioembolization for right-sided, multifocal HCC. MRI can visualize (**A**) tumor on T2-weighted image of liver with hyperintense HCC lesions (arrow), (**B**) targeting of tumor on PET/MR (arrows), and (**C**) intensity of irradiation of surrounding liver tissue on PET (*).

Radioembolization (RE) using Yttrium-90 (^90^Y) microspheres is widely applied for safe and effective HCC therapy in intermediate and advanced Barcelona clinic liver cancer stages. RE can not only achieve tumor shrinkage with detachment from vital structures, allowing margin free resection^5,6^, but also bears the great potential to significantly induce liver hypertrophy of the untreated left liver lobe, with a maximal increases of 30–50% of the untreated liver being reported, substantially increasing FLR^5,7,8^. To date, the minimal needed FLR for safe hepatectomy is still debated in the literature, but ranges from > 20% in otherwise healthy livers to > 40% in patients with impaired liver function due to liver cirrhosis or cholestasis^9^. Liver function tests such as the Liver maximum capacity (MiMAx) test and hepatobiliary scintigraphy (HBS) further deepen the understanding of liver function before major hepatectomies potentially preventing liver failure^10–12^. Radiation applied to the healthy liver tissue causes periportal fibrosis with the above-mentioned consecutive hypertrophy of the contralateral lobe (Fig. 1). Furthermore, a relation was noted between higher radiation dose to the non-tumorous liver tissue of the treated lobe and stronger hypertrophy of the contralateral lobe^13^. PET/MR after RE uses positron emissions of ^90^Y to visualize tumor (diagnostic; Fig. 2A), targeting of tumor (therapy control; Fig. 2B), and intensity of irradiation of the surrounding liver tissue (Fig. 2C).The main challenge in aiming for curative intent RE is the difficulty in predicting volume response after RE, making it only a vague promise for both surgeons and patients.

A preceding study identified several clinical predictive factors, such as absence of ascites, lower Child Pugh Score, small spleen volume, and platelet count ≥ 100/nl, to be associated with increased left liver hypertrophy in patients with HCC treated with right lobe RE^14^. Yet, based on these single values alone, reliable prediction of FLR to estimate the chances of conversion from unresectable to resectable HCC is limited.

Radiomics and clinomics are developments that use advanced machine learning methods to develop computerized models, e.g., for disease prognosis or treatment response based on existing radiological, clinical, or laboratory data^15–17^.

**Figure 3:**
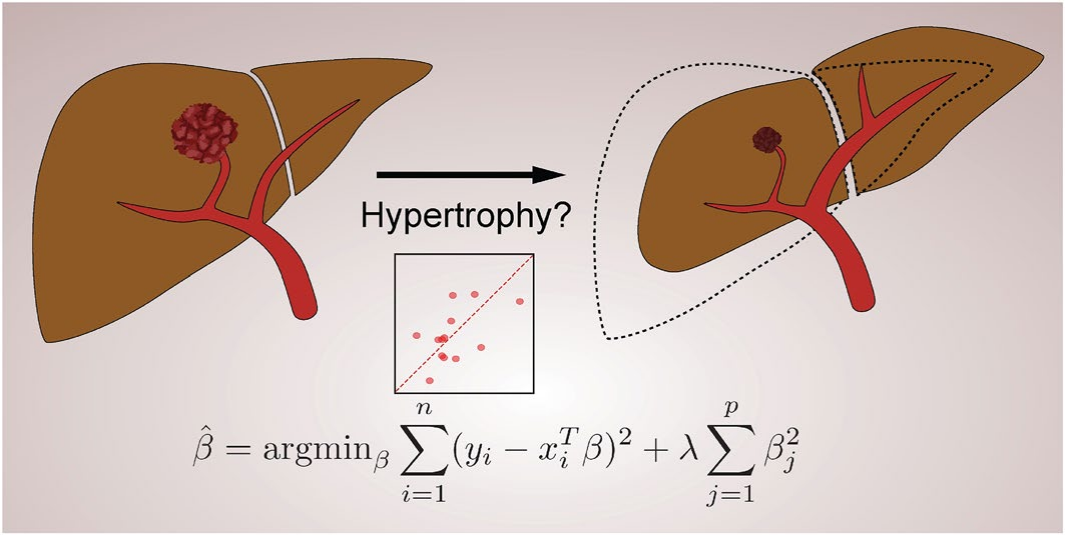
Symbolic visualization of a liver with right sided HCC (left) after RE (right). While tumor necrosis and shrinkage are accompanied by volume reduction of the right lobe, the left lobe hypertrophies. The amount of volume gain of the left liver lobe should be predicted by a machine learning algorithm.

The aim of this study was to utilize machine learning for predicting left liver lobe hypertrophy in patients with HCC and cirrhosis scheduled for right sided RE, with external validation (Fig. 3).

## Results

**Figure 4:**
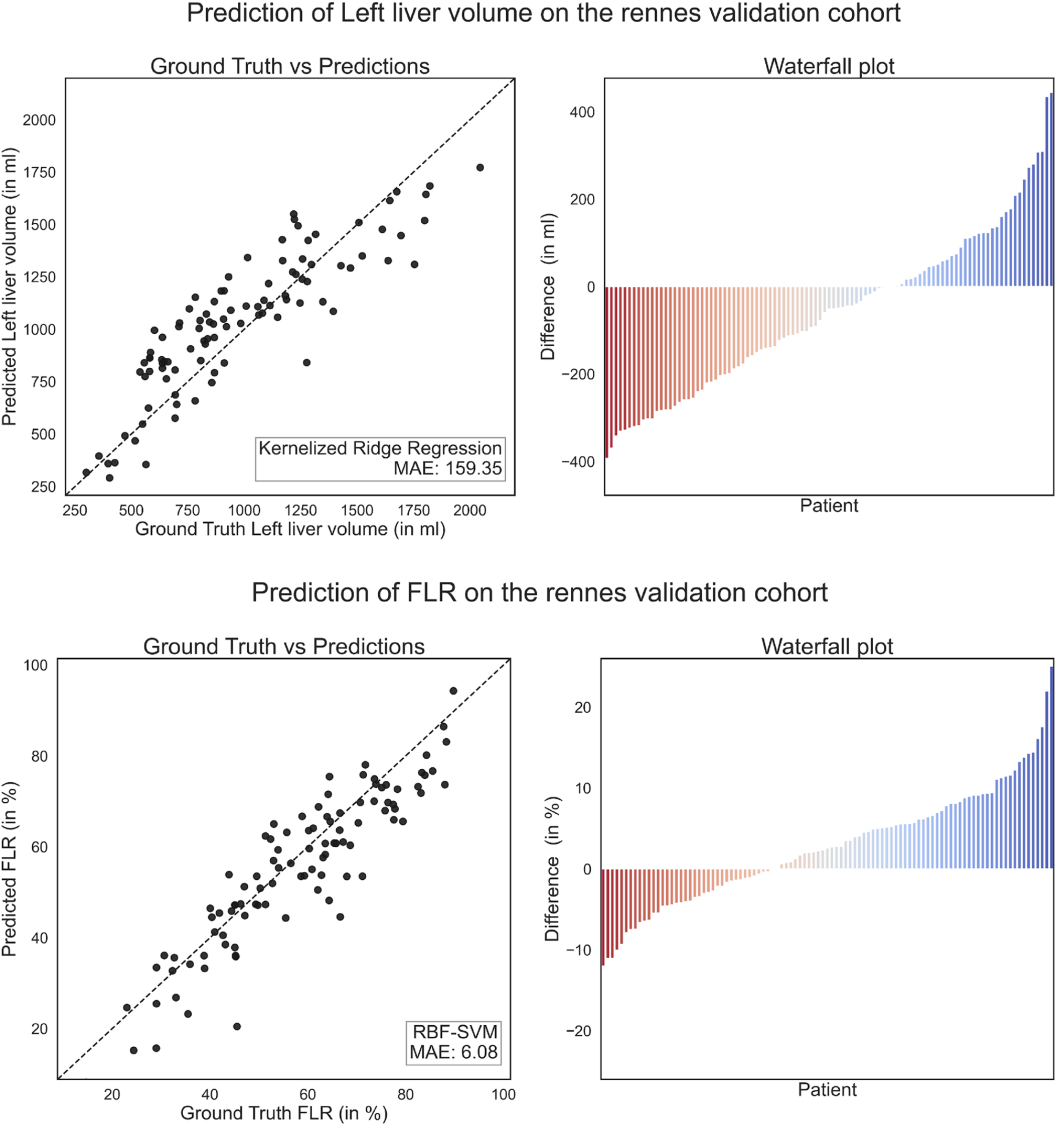
Scatter and waterfall plots illustrate the relative errors of left liver lobe volume predictions (in %) and the absolute errors of the FLR compared to the true liver volume and FLR utilizing the ridge regression model and the RBF-SVM, respectively. Left liver volume in the Rennes validation cohort (top) and FLR in the Rennes validation cohort (bottom).

**Figure 5:**
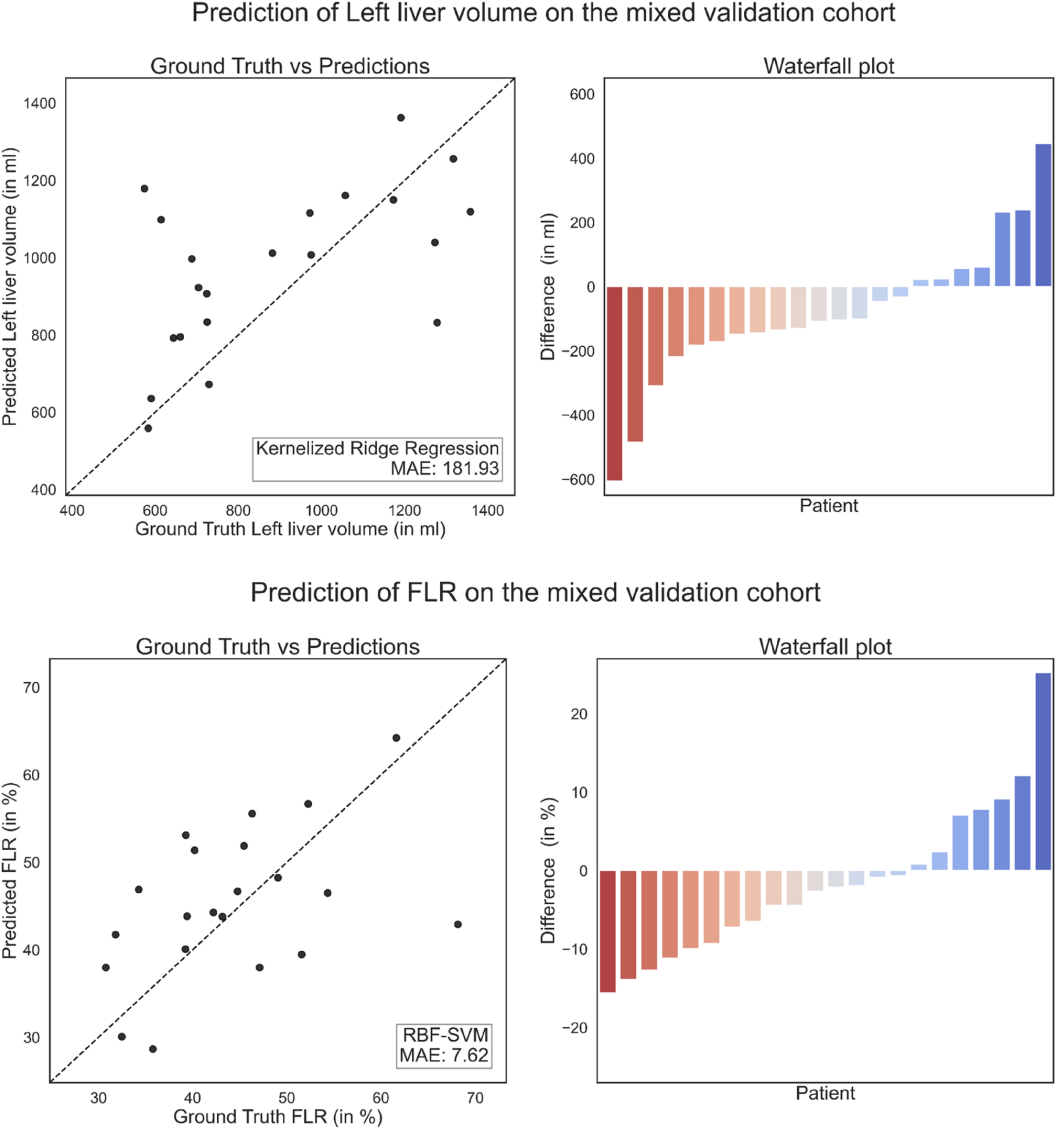
Scatter and waterfall plots illustrate the relative errors of left liver lobe volume predictions (in %) and the absolute errors of the FLR compared to the true liver volume and FLR utilizing the ridge regression model and the RBF-SVM, respectively. Left liver volume in the mixed validation cohort (top) and FLR in the mixed validation cohort (bottom).

High correlations between predicted and measured volumes or FLR were seen during training and internal validation. External validation in the Rennes cohort was excellent (Fig. 4), while correlation in the mixed cohort was less impressive (Fig. 5).

### Predicted liver volume changes in the validation set

In absolute volume prediction, the radial basis function kernelized support-vector machines (RBF-SVM) performed best, showing an absolute mean error of 147.34 ml (95% CI 140.8–153.9 ml) (Supplemental Table 1). For the validation cohorts, the average absolute mean error of the model predictions for left lobar liver volume was 159.4 ml (95% CI 136.8–181.9 ml) and 181.9 ml (95% CI 112.8–251.1 ml) for the cohort from Rennes and the mixed cohort, respectively (Table 1). The distribution of the deviations is visualized in a waterfall plot for both validation cohorts (Figs. 4 and 5).

**Table 1:** Absolute and relative prediction errors of left lobe liver volume predictions measured by computing the mean absolute error (MAE) for the two validation cohorts Rennes and mixed.

		Rennes cohort	Mixed cohort
MAE of the absolute left liver volume in ml (95% CI)	RBF-SVM	159.4 (136.8–181.9)	181.9 (112.8–251.1)
MAE of the FLR in % (95% CI)	Ridge regression	6.08 (5.15–7.82)	7.62 (4.96–10.29)

For relative volume prediction of the FLR, the ridge regression showed the highest precision with an error of 4.97% (95% CI 4.82–5.12%). The prediction of the FLR yielded an error of 6.08% (95% CI 5.15–7.82%) for the cohort from Rennes and similarly 7.62% (95% CI 4.96–10.29%) for the mixed cohort (Table 1); a waterfall plot was again used for visualization (Figs. 4 and 5).

A total of 110 measurements were observed from 49 patients in the Rennes cohort and 22 measurements from 22 patients in the mixed cohort. Eleven measurements from 11 patients in the Rennes cohort occurred later than 9 months after baseline and were therefore removed from the validation set, leaving 99 measurements in this cohort. The median follow-up times was 3.5 months (range, 1.0–9.0 months) and 2.5 months (range, 1.2–8.1 months) for the Rennes and the mixed cohort, respectively.

When predictions were computed with a linear interpolation instead of a natural spline, the MAE of the left liver lobe volume for the Rennes cohort was 153.9 ml. Prediction of FLR yielded an error of 5.95%. Similarly, for the mixed cohort, the prediction error for left lobar volume was 171.4 ml, whereas prediction of FLR showed an error of 7.25%.

### Feature importance in the training set

Logically, permutation importance indicated that all liver volumes at baseline were most important for predictive accuracy (Supplemental Fig. 1). However, their importance diminishes with time. For the prediction of the left lobar volume, age, albumin value, spleen volume, and the presence of a portocaval shunt seem to gain increasing importance over the course of 6 months. Presence of ascites or cirrhosis, Child–Pugh score, thrombocyte count, INR, bilirubin, and sex were of insignificant importance.

### Predicted liver volume changes in the training set

High correlations between predicted and measured volumes or FLR were already eminent during internal validation (Supplemental Table 2).

Predicting the left lobar liver volume, the RBF-SVM performed best and yielded an overall MAE of 147.34 ml (95% CI 140.8–153.9 ml). At each follow-up, performance was worse at later follow-up: The observed MAEs were 87.64 ml (1 month), 132.1 ml (3 months), 169.0 ml (6 months) and 200.6 ml (9 months). The second best model class was XGBoost, which performed only slightly worse with an overall MAE of 151.1 ml (95% CI 144.3–157.9 ml).

For the prediction of FLR, the best performance was obtained by ridge regression, which yielded an overall FLR error of 4.97% (95% CI 4.82–5.12%), with a similar drop in performance over follow-up times (1 month: 2.66%, 3 months: 4.14%, 6 months: 6.14%, 9 months: 6.93%). The RBF-SVM was close to this performance with an overall error of 5.1% (95% CI 4.94–5.26%).

The performance of each model class was tested by 5-fold cross-validation with 20 repetitions on the training set. The smallest mean absolute error in predicting absolute and relative left lobar liver volume was shown by the kernelized ridge regression model.

### Observed liver volume changes

After RE of the right liver lobe, a decrease in volume of the right liver lobe and an increase in volume of the left liver lobe were observed (Fig. 1, Supplemental Table 3). A mean increase of 51.7 ml per 30 days was observed in the training cohort, whereas the increase in the Rennes validation cohort was 49.8 ml per 30 days and similarly 47.3 ml per 30 days in the mixed cohort. A *t* test indicated no significant differences between these increases (all p > 0.10). There was no significant difference in FLR increases per 30 days (2.7% for the training cohort, 3.5% and 2.7% for the Rennes and mixed cohorts, respectively) between the training and the mixed cohort (p > 0.10) and between the mixed cohort and the Rennes cohort (p > 0.10), but there was a significant difference in FLR increases per 30 days between the training and the validation cohort.

### Patient baseline characteristics

Patient characteristics of the different cohorts were compared (Table 1). A total of 146 patients were included in this analysis, contributing 531 volumetry sets for training and validation. Patients in the training cohort had a mean age of 67.3 ± 9.1 years (60 males, 15 females), and 67.9 ± 9.4 years (p = 0.73, 42 males, 7 females) and 63.9 ± 10.6 years (p = 0.18, 17 males, 5 females) in the Rennes and mixed cohort, respectively.

The mean baseline left liver volume in the training cohort was 631 ml (range, 176–1187 ml) with a mean FLR of the left liver lobe of 36% (SD ± 12%). However, these values were significantly higher in the Rennes validation cohort with a median left liver volume of 829 ml (range, 144–1682 ml, p < 0.001) and FLR of 46% (SD ± 15%, p < 0.001). In the mixed cohort, left liver volume was slightly higher compared to the training cohort (750 ml, range, 371–1213 ml, p = 0.042), while FLR was very similar at 36% (SD ± 8%, p = 0.857).

There were more cirrhotic patients in the Rennes cohort (96%, 47/49) than in the training cohort (73%, 55/75; p = 0.003), but none with ascites (p < 0.001). The Child–Pugh score showed a statistically significant difference between the training cohort and the cohort from Rennes (p = 0.045). The number of patients with a portocaval shunt was higher in the training cohort (N = 24) than in the two validation cohorts (N = 2 and N = 1, p < 0.001 and p = 0.021, respectively). Whereas the number of patients with a portal vein thrombosis was significantly higher in the Rennes cohort (N = 23, p < 0.001) than in the training (N = 8) and the mixed cohort (N = 3).

## Discussion

Reliable prediction of left sided hypertrophy after right sided RE has shown to be possible utilizing machine learning algorithms. If the expected volume gain of the left liver is large enough, a sequential curative resection (i.e., major hepatectomy) can be attempted for from the beginning of HCC therapy. While this is one of the most valuable curative treatment options, it is only available to a small percentage of patients due to the risk of post major hepatectomy liver failure with a small FLR^1,5,18,19^. Thus, RE may serve as a conversion therapy from initially unresectable to resectable HCC. The prediction showed a stronger correlation for the Rennes cohort than the mixed cohort. This could be due to a larger population size (49 vs. 22 patients), a higher homogeneity of patients from a single center rather than two separate centers, or the use of a different RE material (glass microspheres for the training and Rennes cohort vs. resin microspheres for the mixed cohort).

Feature importance analysis confirmed some of the features known to correlate with hypertrophy from several preceding studies as part of the machine learning algorithm^6,8,14^. While liver volume at baseline, age, albumin level, spleen volume, and existence of a portocaval shunt had an impact on the prediction of absolute liver volume, these results should be taken with caution as only the univariable importance was analyzed and the interaction between the features were not taken into account.

Of the four different machine learning models evaluated, no single model class of model consistently performed better or worse than the others, underlining the fact that the performance of machine learning models is highly dependent on the data set. In addition, when considering the difference in prediction of the left liver volume in the training cohort between the best and the worst model, a difference of 10.6 ml (147.4 ml vs. 157.9 ml) and 0.6% (4.97% vs. 5.57%) for absolute liver volume and FLR are both too small to be of real clinical importance.

Natural splines were chosen to interpolate the predictions for the validation cohorts as they model biological processes better than several other interpolation methods, since their first and second derivatives are continuous. To understand the extent to which this choice affected the results, standard linear interpolation was also employed to the validation cohort. The only slightly better results for absolute predictions (decrease in error of 5.54 ml (153.9 ml vs. 159.4 ml) and 10.5 ml (171.4 ml vs. 181.9 ml) for the prediction of absolute left lobar volume in the validation and mixed cohort) and relative predictions (FLR differences in favor of linear interpolation by 0.13% and 0.37%, respectively) are not of clinical relevance and do not constitute an argument to dismiss the choice of natural splines.

Simultaneous tumor treatment and hypertrophy induction by RE is a relatively new idea and has to sustain in a world where surgeons have been accustomed to PVE as the standard for inducing liver hypertrophy for over 30 years. It is known that PVE achieves faster growth of FLR and thus faster resectability. However, this is at the risk of tumor progression between PVE and planned resection, resulting in non-resectability in approximately 8% of patients^3,8,20,21^. This risk can be reduced by more accelerated hypertrophy, combining PVE and liver venous deprivation, or sequential TACE of the HCC^4^. In the end, the question remains whether a longer time to possible resection after RE^8^ might be beneficial. Considering that after major hepatectomy intrahepatic tumor recurrence occurs in around 25–41% within one year, it questions the benefit of resection in this population at all^22,23^. Thus, it may be hypothesized that the prolonged time between RE and major hepatectomy may serve as a biological selection tool (“test-of-time”) sorting out patients who would experience early tumor recurrence limiting benefit from surgery. Taken together, if RE is likely to achieve hypertrophy of the untreated left liver lobe with sufficient FLR according to machine learning algorithms, RE may be preferred over PVE due to its direct treatment and potential benefit for conversion therapy.

Liver function tests can evaluate liver function as a whole, i.e. using the LiMAx test, or projected onto anatomical regions or liver segments, i.e. utilizing the HBS^10–12^. The LiMAx test provides additional data on the overall enzymatic liver function by measuring the cytochrome P450 system directly. This proved advantageous over measuring secondary effects of liver function deterioration by means of reduced protein synthesis or reduced detoxification leading to hepatic encephalopathy. HBS on the other hand can precisely measure hepatobiliary excretion per defined liver volume and can thus reduce the risk of failure after major hepatectomies compared to volumetric assessment alone^12^. While an addition of HBS data to our study would be of great value, these are not available since they are not routinely acquired in our institution. HBS has the potential to differentiate volume gain from function gain few days after patients had undergone the ALPPS (associating liver partition and portal vein ligation for staged hepatectomy) procedure, showing that volume gain precedes function gain^24^. A discrepancy between relative liver function and volume was reported by Guiu et al., with the potential to overestimate FLR based on volumetric data alone^25^. We expect volume and function gain few months after RE to be of comparable growth. But neither this, nor the predictive value of baseline HBS of the FLR influencing its hypertrophy potential, have been analyzed so far.

Several machine learning models were explored during training to ensure that the best model was used for prediction since it is generally not known a priori which model will yield the best overall performance. Training proceeded, therefore, by nested cross-validation to identify the best-performing model. In general, extensive exploration of multiple models could lead to overfitting. However, it turned out that in our data, there is no large difference between the models on the training data. Also, no large drop in performance could be seen when considering the validation cohort from Rennes. Both indicate that the selected model does not overly overfit the data. Nonetheless, slightly larger differences were seen for the mixed validation cohort; this might show that the model depends strongly on baseline characteristics or possibly the device used for RE (resin vs. glass microspheres), which were different in that cohort. The applicability to other patient cohorts may thus be limited.

In addition, the sample sizes for the training and validation cohorts were rather small. In general, small sample sizes can potentially lead to false-positive findings, yet, our model was tested on two external validation sets with good results, demonstrating that the model can generalize. While larger sample sizes are warranted to improve accuracy and ideally allow for an application in clinical routine, the availability of such data sets is currently limited. Future studies with more patients included, ideally from different populations, must be conducted before such models are introduced in the clinical routine.

Additionally, data on radiation dose to the non-tumorous liver tissue was not taken into account, despite indications that hypertrophy of the untreated lobe depends on this^13^. Dose data will be important to include in future studies. Importantly, the biological effects of the two devices used (glass and resin microspheres) will be different and must be considered: radiation activity per microsphere varies (glass microspheres higher than resin), and different distribution patterns due to varying numbers of particles (resin higher numbers than with glass) can be expected.

Furthermore, as the study population consisted of patients with HCC exclusively of the right liver lobe suitable for RE, transferability to patients with metastases of the liver (e.g., colorectal cancer), patients not eligible for RE, patients with worse liver function and tumors of the left liver lobe or both liver lobes only may be limited, potentially warranting specific further evaluation for these patient populations. Finally studies with a prospective nature should be performed to further validate such algorithms.

In conclusion, we proposed a machine learning algorithm to accurately predict relative and absolute volume changes of the left liver lobe after right-sided RE. External validation confirmed the functionality of the prediction algorithm, which could help estimate the chances of conversion from palliative RE to curative hepatectomy following significant FLR hypertrophy.

## Methods

### Patients

This multi-center retrospective study was conducted in accordance with the Declaration of Helsinki and approved by the Ethics Commission of the Medical Faculty of the University of Duisburg-Essen (17-7754_2-BO), and is a succeeding study of a patient cohort previously reported^14^ used as training cohort in this study. Informed consent for all procedures was given by all patients. Ethics commissions of the three external sites also approved usage of the data (Münster: ethics approval 2018-638-f-S) or waived need for additional approval due to the retrospective nature of the study (University of Würzburg, Centre Eugène Marquis, Rennes). HCC patients treated with RE were collected from four different sites: 75 patients treated between January 2007 and April 2015 from the University Hospital Essen, Germany were included, which comprised the training cohort. In addition, 49 patients treated between November 2009 and March 2016 from the University Hospital Rennes, France were used as a first validation cohort, and 17 patients treated between August 2012 and December 2017 from the University Hospital Münster, Germany, as well as 5 patients treated between June 2010 and December 2014 from the University Hospital Würzburg, Germany, which were used collectively in a second validation cohort. Overall, half of the patients underwent liver biopsy for diagnosis, while the remaining patients were diagnosed based on imaging alone. Treatment indications for most patients were unresectability, size (treatment alternative to TACE), and 10% bridge to liver transplantation.

Inclusion criteria were (a) RE only to the right liver lobe for unresectable unilateral HCC, (b) computed tomography (CT) imaging or magnetic resonance imaging (MRI) of the liver with a maximum of 6 weeks prior to RE and at least one follow-up imaging at least 1 month thereafter. Additional inclusion criteria for the Essen cohort was imaging follow-up at 3, 6 and 9 months. Exclusion criteria were (a) previous segmentectomy or surgical liver resection, and (b) previous transarterial chemoembolization procedure within 12 months prior to RE.

Patients in the training cohort from Essen and the validation cohort from Rennes were treated with glass ^90^Y microspheres, while the other two cohorts from Münster and Würzburg were treated with resin ^90^Y microspheres.

### CT scans

Volumetric analysis was performed on baseline CT or MRI scans and on the corresponding follow-up scans in the portal phase. The classification of hepatic segments followed the Couinaud segmentation, if required adjusted by SPECT information gathered during the planning phase^26^. Volumetry was performed with site specific techniques, but all were performed on modern CT scanners with 3–5 mm thick reconstructed slices with manual summation of all slices or automatic calculation on a Syngo data-processing console display unit (Siemens Healthineers, Erlangen, Germany). Volumetry before and after treatment was performed by an identical radiologist or nuclear medicine physician with at least 5 years of experience. Three volumes were measured: Left lobe, right lobe and total volume (all in ml). From these, the FLR was computed as the ratio of left lobe volume to the total volume (in %). Additionally, the total splenic volume was measured at baseline.

In the training cohort, imaging scans were taken at 3, 6 and 9 months after RE. A few patients (N = 14) were missing scans at 9 months after RE. For these, the liver volume at 9 months was estimated by computing the mean difference between the 6 month and the 9 month follow-up values over all other patients and adding this difference to the 6 month follow-up to obtain an estimate for the 9 month follow-up.

Imaging scans in the validation cohorts were taken at different follow-up time points (i.e., 4 or 7 months), and up to three follow-up measurements were available per patient.

### Patient characteristics

Baseline characteristics of patients, including laboratory results were extracted from chart review (Supplemental Table 4). Laboratory features were prothrombin time (international normalized ratio; INR), albumin, thrombocyte count and total bilirubin. Spleen volume was measured at baseline. Collected clinical features were sex, etiology of cirrhosis, Child–Pugh score, ascites, portocaval shunting on initial cross-sectional imaging and portal vein thrombosis. If no cirrhosis was found, the Child–Pugh score was not defined and was set to zero. A summary of the characteristics of the features from the three cohorts is displayed (Supplemental Table 4). From the radiological imaging, liver volume (left lobe [ml], right lobe [ml], total liver [ml], FLR = left/total [%]) and total splenic volume (ml) before RE were used. Clinical characteristics were complete for all patients. All features except for the baseline volumes were scaled to have a mean of 0 and a variance of 1. Baseline volumes were expressed in liters to ensure that all features have a similar range.

### Machine learning analysis

Machine learning can be considered as a subdomain of statistics, with the aim of finding predictive models that learn the underlying relationship in a given data set between the predictors and endpoints. Their advantage is that they learn this relationship in a data-driven way. Potentially, machine learning models are complex enough to memorize the data, which affects the model's ability to generalize to new, unseen data. Therefore, validation methods must be used to estimate the performance of a model.

Several model classes were used to analyze the data: Linear and kernelized support-vector machines (SVM), kernelized ridge regression^27^, which is a well-known non-linear variant of least square regression with an additional regularization term as well as XGBoost^28^, an optimized variant of gradient boosting. For the non-linear models, the radial basis function kernel was employed. Prediction error was measured by computing the mean absolute error (MAE).

### Training

Models were trained to predict FLR and absolute left lobar volumes using only baseline characteristics, i.e., clinical features, laboratory features and spleen and liver volumes before RE.

Repeated double-cross validation was used to build and estimate the performance of the models as outlined in Supplemental Fig. 2^29^. In the outer cross-validation step, the data was split randomly into five folds. Four of these were used in an inner 5-fold cross-validation step to find the best parameter of the corresponding model, and its performance was assessed on the remaining fold. This procedure was repeated twenty times and final estimates were obtained by averaging the resulting MAEs. In the inner cross-validation step, the data was split again in five folds and the hyper-parameters of the models were optimized. Both SVMs were optimized using the epsilon-insensitive loss, while for the XGBoost regression the mean squared loss was employed.

Independent models for each model class were trained in this fashion to predict the absolute left lobar volume and FLR at 1, 3, 6 and 9 months after baseline, respectively. The model class with the lowest average MAE was selected as the final model class and retrained over the whole training set. The hyper-parameters for the final model were selected by 5-fold cross-validation.

To understand the relative importance of the included features, their univariable importance was calculated during cross-validation using the permutation importance method^30^. To this end, for all models trained during cross-validation, the evaluation on the corresponding test data was modified as follows: First, the accuracy of the model was determined on the test data. Then, for each feature, its values in the test data were permuted and the model was re-evaluated. The difference in accuracy between the base model (with all features) and the model with the permuted feature was calculated. This difference represents the loss in accuracy when permuting the corresponding feature and gives a rough estimation of its relative importance in the trained model. The overall importance of the features was then obtained by averaging. Because four different models were trained for 1, 3, 6, and 9 months, this approach provides the feature importance for each model and shows how the relative importance of a feature changes through time.

### Validation cohorts

**Figure 6:**
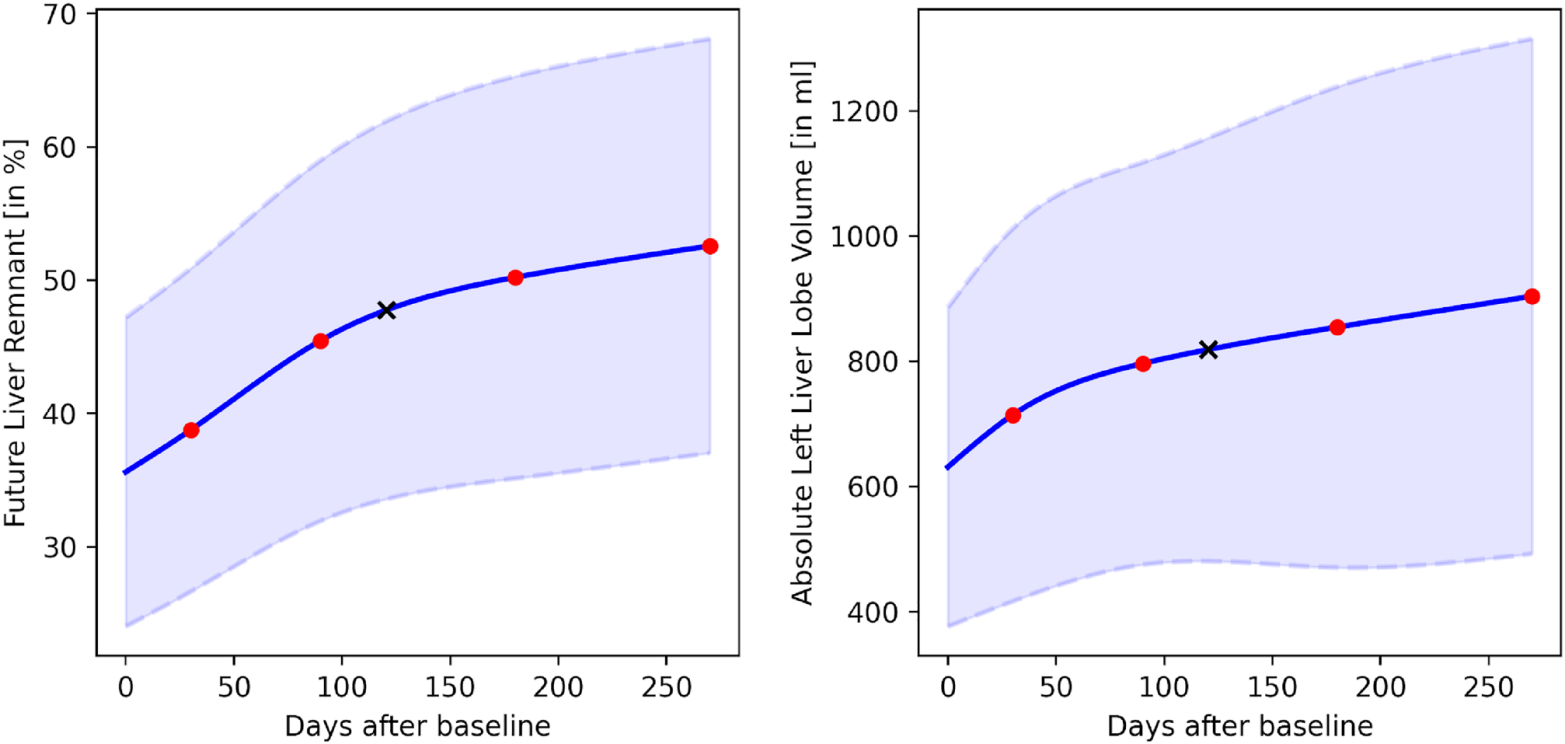
Mean and standard deviation of the FLR (left) and the absolute liver volume (right) on the training cohort. The values were computed at the measurements at 1, 3, 6 and 9 months after RE (red dots), while the curves were interpolated using natural splines, e.g. the estimate of the mean liver volume at 4 month (black cross) was computed.

Patients in the two validation cohorts were measured at different time points, e.g., at 2, 3.5 and 5 months after baseline and were thus not directly comparable to measurements of patients in the training set. Therefore, the following procedure was applied: First, as the training set did not contain any measurements beyond 9 months after RE, such measurements were removed from the validation set (N = 9). Then, the trained models were used to predict the liver volumes or FLR at 1, 3, 6 and 9 months after baseline. To obtain measurements at the times when measurements were available for the patients in the validation set, natural splines were used to interpolate the predicted values^31^. This allowed prediction of the volume or FLR at any time after baseline up to 9 months (Fig. 6). Natural cubic splines were chosen for interpolation as they are smooth and model the underlying biological process better than simple linear interpolation, which introduces sharp changes. However, in order to understand how this choice affects the results, linear interpolation was also used strictly for reasons of comparison.

The overall error for the validation cohorts was obtained by averaging all absolute differences between the predicted and the true volume or FLR for each patient. In case multiple follow-up measurements were available for a patient, a volume or FLR was predicted for each time point and the resulting errors were then averaged to obtain a single error.

Baseline clinical data is either stated in percentage or mean values with standard deviation (SD) if not stated otherwise. Machine learning modelling was performed by using the scikit-learn package of python 3.7. Two-sided *t* tests and Chi-square tests were calculated using R 3.6.1.

## Supplementary Information

Supplementary Information.

## Data Availability

The datasets used and analyzed are available from the corresponding author on reasonable request due to data protection protocol of the institutions.

## Abbreviations

BCLC: Barcelona clinic liver cancer
CI: Confidence interval
CT: Computed tomography
INR: International normalized ratio
FLR: Future liver remnant
HCC: Hepatocellular carcinoma
MAE: Mean absolute error
PVE: Portal vein embolization
RE: Radioembolization
RILD: Radiation induced liver damage
SD: Standard deviation
RBF-SVM: Radial basis function kernelized support-vector machines
^90^Y: Yttrium-90

## References

[1] Cauchy F, Soubrane O, Belghiti J (2014). Liver resection for HCC: Patient’s selection and controversial scenarios. Best Pract. Res. Clin. Gastroenterol..

[2] Kubota K (1997). Measurement of liver volume and hepatic functional reserve as a guide to decision-making in resectional surgery for hepatic tumors. Hepatology.

[3] Aoki T, Kubota K (2016). Preoperative portal vein embolization for hepatocellular carcinoma: Consensus and controversy. World J. Hepatol..

[4] Yoo H (2011). Sequential transcatheter arterial chemoembolization and portal vein embolization versus portal vein embolization only before major hepatectomy for patients with hepatocellular carcinoma. Ann. Surg. Oncol..

[5] Cucchetti A (2016). Selective internal radiation therapy (SIRT) as conversion therapy for unresectable primary liver malignancies. Liver Cancer.

[6] Vouche M (2013). Radiation lobectomy: Time-dependent analysis of future liver remnant volume in unresectable liver cancer as a bridge to resection. J. Hepatol..

[7] Theysohn JM (2014). Hepatic volume changes after lobar selective internal radiation therapy (SIRT) of hepatocellular carcinoma. Clin. Radiol..

[8] Garlipp B (2014). Left-liver hypertrophy after therapeutic right-liver radioembolization is substantial but less than after portal vein embolization. Hepatology.

[9] Guglielmi A, Ruzzenente A, Conci S, Valdegamberi A, Iacono C (2012). How much remnant is enough in liver resection?. Dig. Surg..

[10] Stockmann M (2009). Prediction of postoperative outcome after hepatectomy with a new bedside test for maximal liver function capacity. Ann. Surg..

[11] Ekman M, Fjälling M, Friman S, Carlson S, Volkmann R (1996). Liver uptake function measured by IODIDA clearance rate in liver transplant patients and healthy volunteers. Nucl. Med. Commun..

[12] de Graaf W (2010). Assessment of future remnant liver function using hepatobiliary scintigraphy in patients undergoing major liver resection. J. Gastrointest. Surg..

[13] Palard X (2018). Dosimetric parameters predicting contralateral liver hypertrophy after unilobar radioembolization of hepatocellular carcinoma. Eur. J. Nucl. Med. Mol. Imaging.

[14] Goebel J (2017). Factors associated with contralateral liver hypertrophy after unilateral radioembolization for hepatocellular carcinoma. PLoS One.

[15] Gillies RJ, Kinahan PE, Hricak H (2016). Radiomics: Images are more than pictures, they are data. Radiology.

[16] Lambin P (2012). Radiomics: Extracting more information from medical images using advanced feature analysis. Eur. J. Cancer.

[17] Parmar C, Grossmann P, Bussink J, Lambin P, Aerts HJWL (2015). Machine learning methods for quantitative radiomic biomarkers. Sci. Rep..

[18] European Association for the Study of the Liver and European Organisation for Research and Treatment of Cancer (2012). EASL–EORTC Clinical Practice Guidelines: Management of hepatocellular carcinoma. J. Hepatol..

[19] Kim HJ (2013). Comparison of remnant to total functional liver volume ratio and remnant to standard liver volume ratio as a predictor of postoperative liver function after liver resection. Korean J. Hepatobiliary Pancreat. Surg..

[20] Pamecha V (2009). Effect of portal vein embolisation on the growth rate of colorectal liver metastases. Br. J. Cancer.

[21] Marti J (2018). Analysis of preoperative portal vein embolization outcomes in patients with hepatocellular carcinoma: A single-center experience. J. Vasc. Interv. Radiol..

[22] Cheng Z (2015). Risk factors and management for early and late intrahepatic recurrence of solitary hepatocellular carcinoma after curative resection. HPB.

[23] Siriwardana RC, Lo CM, Chan SC, Fan ST (2012). Role of portal vein embolization in hepatocellular carcinoma management and its effect on recurrence: A case–control study. World J. Surg..

[24] Olthof PB (2017). Hepatobiliary scintigraphy to evaluate liver function in associating liver partition and portal vein ligation for staged hepatectomy: Liver volume overestimates liver function. Surgery.

[25] Guiu B (2021). 99mTc-mebrofenin hepatobiliary scintigraphy and volume metrics before liver preparation: Correlations and discrepancies in non-cirrhotic patients. Ann. Transl. Med..

[26] Strasberg SM, Phillips C (2013). Use and dissemination of the Brisbane 2000 nomenclature of liver anatomy and resections. Ann. Surg..

[27] Cristianini N, Shawe-Taylor J (1999). An Introduction to Support Vector Machines: And Other Kernel-Based Learning Methods.

[28] Chen, T. & Guestrin, C. XGBoost: A scalable tree boosting system. in *Proceedings of the 22nd ACM SIGKDD International Conference on Knowledge Discovery and Data Mining* 785–794 (ACM Press, 2016). 10.1145/2939672.2939785.

[29] Filzmoser P, Liebmann B, Varmuza K (2009). Repeated double cross validation. J. Chemom..

[30] Breiman L (2001). Random forests. Mach. Learn..

[31] Birkhoff G, Garabedian HL (1960). Smooth surface interpolation. J. Math. Phys..

