# Supplementary material for: Prediction of left lobe hypertrophy after right lobe radioembolization of the liver using a clinical data model with external validation
Source: Sci Rep. 2022 Dec 1;12:20718. doi: 10.1038/s41598-022-25077-6 (PMC9715713; doi:10.1038/s41598-022-25077-6)
Supplement: Supplementary file 1 — Supplementary Information. [file 41598_2022_25077_MOESM1_ESM.pdf]

Supplementary information files

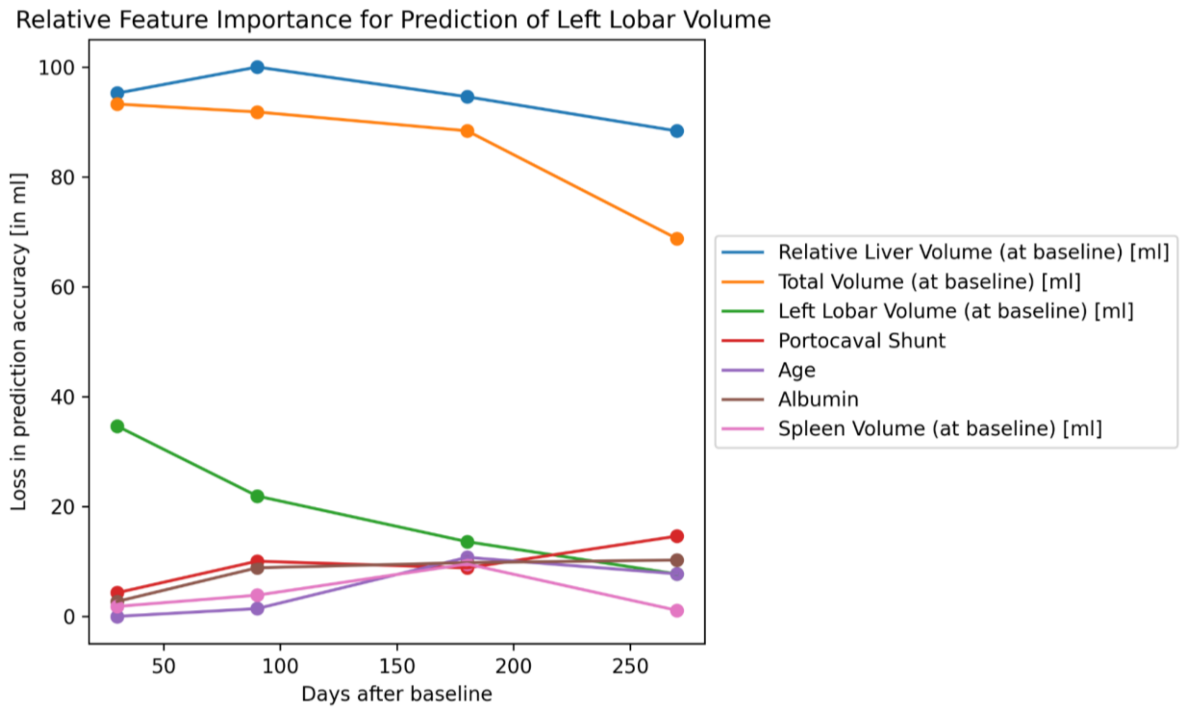

Supplemental Figure 1. Relative univariate feature importance computed using permutation importance method for the prediction of left lobar volume. The six most important features are shown.

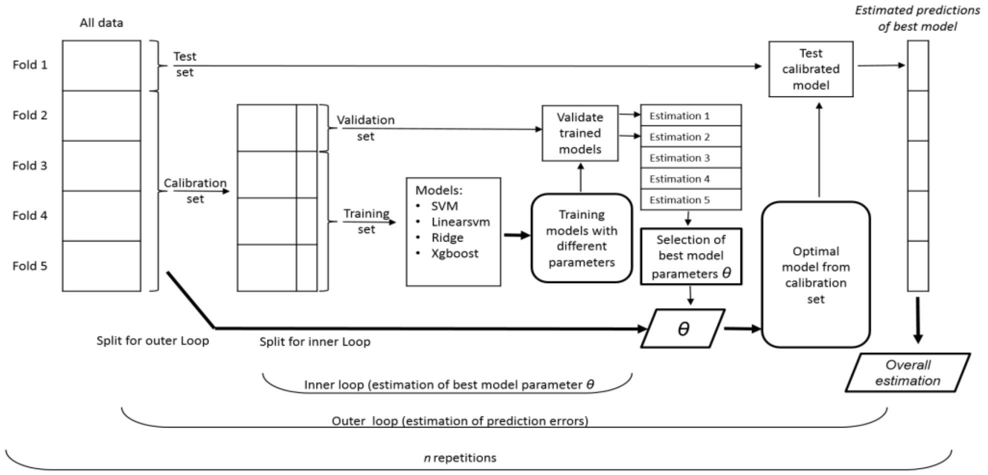

Supplemental Figure 2: Repeated double-cross validation. Data was split randomly into five folds. Four of these were used to find the best parameter of the corresponding model (SVM, linear SVM, ridge regression, XGBoost). Their performance was assessed on the remaining fold.

Supplemental Table 1. Optimized hyper-parameters during the cross-validation.

Model class	Parameter	Range
Linear SVM	C	2^-16 .. 2^16
	□	2^-16 .. 2^0
RBF-SVM	C	2^-16 .. 2^16
	□	2^-16 .. 2^16
	□	2^-16 .. 2^0
Ridge Regression	□	2^-16 .. 2^16
	□	2^-16 .. 2^16
XGBoost	colsample_bytree	0.5, 0.6, 0.7, 0.8, 0.9, 1.0
	subsample	0.8, 0.9, 1.0
	max_depth	2, 3, 5, 7
	gamma	0, 1, 3, 5
	n_estimators	25, 50, 125, 250
	learning_rate	0.01, 0.025, 0.05, 0.1, 0.25

Supplemental Table 2: Absolute and relative prediction errors of left lobe liver volume predictions in the training set (Essen), measured by computing the mean absolute error (MAE). The overall average is for comparison purposes only. Best values are marked in bold. 95%CI, 95% Confidence interval; FLR, future liver remnant.

		1 month after baseline	3 month after baseline	6 month after baseline	9 month after baseline	Overall Average
MAE of the absolute left liver volume in ml (95% CI)	Ridge Regression	98.84 [93.93, 103.76]	133.8 [127.67, 139.93]	185.16 [176.4, 193.92]	213.79 [203.68, 223.9]	157.9 [151.26, 164.54]
	RBF-SVM	**87.64** [82.9, 92.37]	132.14 [126.55, 137.73]	**169.02** [160.27, 177.77]	200.56 [189.8, 211.32]	**147.34** [140.83, 153.85]
	XGBoost	96.14 [90.93, 101.34]	**129.33** [123.43, 135.23]	179.01 [169.75, 188.26]	**199.89** [189.15, 210.63]	151.09 [144.27, 157.92]
	Linear SVM	90.94 [86.41, 95.47]	132.87 [126.4, 139.33]	174.95 [165.76, 184.14]	211.61 [200.57, 222.65]	152.59 [145.79, 159.39]
MAE of the FLR in % (95% CI)	Ridge Regression	2.66 [2.54, 2.78]	**4.14** [3.99, 4.29]	**6.14** [5.92, 6.36]	**6.93** [6.66, 7.19]	**4.97** [4.82, 5.12]
	RBF-SVM	2.45 [2.32, 2.58]	4.42 [4.25, 4.59]	6.31 [6.08, 6.55]	7.2 [6.91, 7.5]	5.1 [4.94, 5.26]
	XGBoost	2.82 [2.71, 2.94]	4.87 [4.7, 5.05]	6.96 [6.72, 7.19]	7.65 [7.38, 7.92]	5.57 [5.43, 5.72]
	Linear SVM	**2.43** [2.32, 2.54]	4.69 [4.51, 4.87]	6.65 [6.33, 6.97]	7.55 [7.26, 7.84]	5.33 [5.16, 5.5]

Supplemental Table 3: Observed absolute and relative volumetric liver lobe changes induced by after radioembolization (RE) of the right hepatic lobe in the training cohort.

Time points (months after RE of right liver lobe)	Absolute volume in ml (median & range)		Relative liver volume in % ± SD (range)	
	Right lobe	Left lobe	Right lobe	Left lobe
Baseline	1094 (433 - 2737)	562 (176 - 1187)	64 ± 12 (38 - 90)	36 ± 12 (10 - 62)
1 month	1091 (420 - 3301)	657 (136 - 1453)	61 ± 12 (36 - 92)	39 ± 12 (8 - 64)
3 months	917 (276 - 2660)	727 (205 - 1602)	55 ± 14 (25 - 86)	45 ± 14 (14 - 75)
6 months	777 (126 - 2526)	794 (221 - 2018)	50 ± 15 (13 - 81)	50 ± 15 (19 - 87)
9 months	713 (214 - 2778)	806 (274 - 2373)	47 ± 16 (21 - 80)	53 ± 16 (20 - 79)

Supplemental Table 4: Patient characteristics at baseline for the three cohorts. Values are reported either as percentages for categorical variables or as mean +/- SD for continuous variables. Chi squared tests and two-sided t-tests were used to compare the groups respectively.

	**Training Cohort (Essen) (N = 75)**	**Validation Cohort “Rennes” (N = 49)**	**Validation Cohort “Mixed” (N = 22)**	**p-Value (Essen vs Rennes)**	**p-Value (Essen vs Mixed)**
Age [years]	67.27 +/- 9.06	67.86 +/- 9.37	63.86 +/- 10.64	0.729	0.183
Sex [female]	20%	14%	23%	0.566	1.0
Cirrhosis [present]	73%	96%	64%	0.003	0.539
Child Pugh Score<br>5<br>6<br>7	 <br>30<br>16<br>9	 <br>34<br>12<br>1	 <br>9<br>1<br>4	<0.001	<0.001
Ascites [present]	28%	0%	18%	<0.001	0.517
Portocaval shunt [present]	32%	4%	5%	<0.001	0.021
Portal vein thrombosis	11%	47%	14%	<0.001	0.997
Albumin [g/dl]	4.04 +/- 0.52	3.86 +/- 0.49	3.81 +/- 0.74	0.05	0.193
Bilirubin [mg/dl]	0.83 +/- 0.49	0.96 +/- 0.5	0.67 +/- 0.28	0.172	0.054
INR	1.07 +/- 0.1	1.13 +/- 0.15	1.09 +/- 0.1	0.014	0.521
Thrombocytes [tsd./ul]	161.68 +/- 91.82	153.49 +/- 63.46	165.55 +/- 68.39	0.558	0.831
Total volume (at baseline) [ml]	1797.29 +/- 506.44	1763.61 +/- 418.28	2108.5 +/- 535.56	0.688	0.021
Left volume (at baseline) [ml]	631.26 +/- 256.31	819.55 +/- 323.13	749.59 +/- 224.48	0.001	0.042
Relative volume= left/total (at baseline) [%]	35.63 +/- 11.65	46.25 +/- 14.58	36.04 +/- 8.45	<0.001	0.857
Spleen volume [ml]	480.4 +/- 338.4	439.02 +/- 249.34	433.59 +/- 251.88	0.435	0.485
